# Paediatric Uveitis - the uniqueness in clinical presentation and the efficacy of biologics treatment

**DOI:** 10.1186/s12348-024-00415-z

**Published:** 2024-07-17

**Authors:** Mei Kwan Yiu, Mary Ho, Assunta C.H. Ho, Winnie K.Y. Chan, Wing Yung, Wilson W.K. Yip, Alvin L. Young

**Affiliations:** 1 Department of Ophthalmology & Visual Sciences, Prince of Wales Hospital, The Chinese University of Hong Kong, 30-32 Ngan Shing Street, Shatin, New Territories, Hong Kong SAR, China; 2 Department of Paediatrics, Prince of Wales Hospital, The Chinese University of Hong Kong, Hong Kong SAR, China; 3 Department of Paediatrics, Queen Elizabeth Hospital, Hong Kong SAR, China

**Keywords:** Paediatric uveitis, Immunosuppressants, Biologics

## Abstract

**Aims:**

To evaluate unique clinical characteristics of paediatric uveitis in our locality and treatment outcomes especially the efficacy of biologics.

**Methods:**

This was a retrospective cohort.

**Results:**

37 paediatric uveitis cases involving 67 eyes were included. Male-to-female ratio was 1:1.3. Mean age of uveitis onset was 11 ± 3.7 (4–18). 81.1% cases suffered from bilateral uveitis. 75.7% cases were chronic uveitis. Nearly half of the cases (40.5%) presented with anterior uveitis. The predominant diagnosis of uveitis in our cohort was idiopathic. Unlike studies from other populations, the associated systemic conditions in this mostly Chinese cohort were Behçet’s disease (8.1%), tubulointerstitial nephritis and uveitis (8.1%) and HLA-B27 associated uveitis (8.1%). Steroid response was a common phenomenon, observed in 40.5% of cases. The most common complication was posterior synechiae (45.9%), followed by cataract (37.8%), glaucoma (27.0%), band keratopathy (18.9%) and macular oedema (13.5%). 3/37 patients encountered either first attack of uveitis or flare after receiving COVID-19 vaccine. 54.1% of patients required systemic steroid for disease control. The majority required steroid sparing immunotherapy, including Methotrexate (43.2%), Mycophenolate Mofetil (24.3%), Cyclosporine A (8.1%), Azathioprine (5.4%) and Tacrolimus (2.7%). Resistant cases required biologics including tumour necrosis factor alpha inhibitors (Adalimumab 32.4%, Infliximab 2.7%) and interleukin-6 inhibitors (Tocilizumab 2.7%).

**Conclusions:**

Clinical presentation of the local paediatric uveitis differs from previously described features in Caucasian and other populations. According to our experience as a tertiary eye centre, Behçet’s disease, tubulointerstitial nephritis and uveitis and HLA-B27 associated uveitis were more often encountered than Juvenile Idiopathic Arthritis associated uveitis. Our report evaluated the efficacy of immunomodulatory therapy and biologics in controlling uveitis and reducing ocular complications.

## Introduction

Paediatric uveitis, a rare and potentially sight threatening condition, poses unique challenges and implications for ophthalmologists. While uveitis can occur across all age groups, its incidence in paediatric population is relatively low, accounting for approximately 5–10% of all uveitis cases [1]. This rarity presents an unmet need in our understanding on paediatric uveitis, in terms of early recognition, accurate diagnosis and appropriate management. Diagnosing paediatric uveitis can be particularly challenging due to its diverse clinical presentations, potential resemblance to other ocular conditions and limited examination in uncooperative children. The management of paediatric uveitis involves choosing appropriate treatment strategies to control inflammation while minimizing potential side effects. However, there is no standardized treatment protocol due to variations in disease severity, aetiology, and patient characteristics [2]. The current available cases series studying paediatric uveitis were mainly published based on the population in the region of North America, Australia and Europe [3–7]. The description on paediatric uveitis in Asian population is very limited. The aetiology and clinical presentation appeared to be different between different ethnic groups and geographic locations. Hence, this study aims to describe the demographics, clinical characteristics and treatment outcomes of paediatric uveitis in a tertiary eye centre in Hong Kong.

## Methods

This is a retrospective cohort study of paediatric uveitis. The cases were identified in a tertiary eye centre from 1/1/2022 to 31/12/2023. This study was conducted in conformance with the ethical principles of the Declaration of Helsinki and the International Conference on Harmonisation Guidelines for Good Clinical Practice. Data on patients’ demographics, medical and ocular history, physical examination findings, investigation results, treatment regimen and treatment response were retrieved from electronic medical records and consultation notes. Patients who first developed uveitis at or below 18 years old were included in our study. The median follow-up period was 84 months, with a minimum duration of 6 months. All patients were managed by ophthalmologists specialized in uveitis. Clinical records from paediatric clinics were retrieved if the patients were followed up by paediatricians for related systemic diseases or usage of systemic immunosuppressants. The classification of uveitis was based on Standardization of Uveitis Nomenclature (SUN) criteria [8] (Table 1). Work up for uveitis included blood tests, especially autoimmune markers (anti-nuclear antibodies ANA and rheumatoid factor RF) and human leukocyte antigen B27 HLA-B27. Optical coherence tomography was performed when there was suspicion of macular oedema. Topical steroid or periocular steroid were initiated by ophthalmologists according to ocular condition. If the patients developed rise in intraocular pressure after local steroid, glaucomatous topical eye drops were given and topical prednisolone acetate was replaced by loteprednol. Systemic immunosuppressants were chosen based on the consensus of both paediatric rheumatologists and ophthalmologists according to disease phenotype, treatment response, patients’ comorbidities and tolerance. Screening for preexisting infections including hepatitis, tuberculosis and conditions with reference to individual risk profiles were carried out to exclude contraindications for systemic immunosuppressants. Oral prednisolone was indicated when there was poor response to topical or local steroid therapy, or in cases of systemic disease manifestation. Poor response was defined as uncontrolled or deteriorating ocular inflammation. We initiated oral prednisolone at a dose of 1 mg/kg/day for severe uveitis or sight-threatening cases, following consultation with a paediatric rheumatologist. It was then tapered gradually once disease was under control. The tapering schedule [9] shown in Table 2. Steroid sparing agents were started as soon as possible for severe (defined as sight threatening with posterior and macular involvement, chronic persistent inflammation for at least 3 months) or refractory (defined as failure of systemic steroid therapy, relapse after reduction of systemic steroid) cases in order to minimize the use of systemic steroid and their side effects. They included antimetabolites (Methotrexate, Mycophenolate Mofetil, Azathioprine), and calcineurin inhibitors (Cyclosporine A, Tacrolimus). The treatment effect of the above first line steroid sparing treatment was observed for at least 3 months.For cases that were either unresponsive or intolerant to the standard first line steroid sparing agents as described above, biologics including tumour necrosis factor-alpha inhibitors (for example, Infliximab, Adalimumab, Golimumab) and interleukin-6 inhibitors (for example, Tocilizumab) were initiated as second line steroid sparing treatments. The adoption of biologics as a second line option was largely influenced by local government funding policies. We opted for adalimumab instead of infliximab primarily due to its route of administration, the availability of funding programs, and the presence of a 20 mg pre-filled syringe suitable for younger paediatric patients. Uveitis was considered under control when there were no active inflammatory lesions, no cells in anterior chamber, no cells in anterior vitreous and no vitritis clinically. Also, the treatment control was achieved when patients experienced no recurrence and maintained completely quiet eyes for three months. For teenage patients of adult weight, this included maintaining an oral steroid dosage of less than 7.5 mg daily. For children, treatment control was defined as being completely free of steroids.Surgical interventions including cataract surgery and glaucoma surgery were performed, when necessary, in treatment of complications of uveitis.

**Table 1:** Classification of uveitis

**Anatomical** :	**Primary site of inflammation** :
Anterior	Anterior chamber
Intermediate	Vitreous
Posterior Panuveitis	Retina or choroid Anterior chamber, vitreous, and retina or choroid
**Duration**:	
Limited	**≤** 3months’ duration
Persistent	**≥** 3months’ duration
**Course** :	
Acute	Episode of sudden onset and limited duration
Recurrent	Repeated episodes separated by periods of inactivity without treatment **≥** 3months’ duration
Chronic	Persistent uveitis with relapse **≤** 3months after discontinuing treatment

**Table 2:** Tapering schedule of Prednisolone

Initial dosage (mg/day)	Decrement dose (mg/day)	Taper interval
60 − 30	10	Weekly
30 − 15	5	Weekly
15 − 7.5	2.5	Weekly
< 7.5	2.5-1	Weekly-monthly

### Statistical analysis

Descriptive data were reported with mean with standard deviation and proportion. Paired-sample Student’s t test was applied to test for differences between the visual acuity at uveitis onset and when uveitis was under control. In cases of bilateral involvement, visual acuity of the worse eye was taken for statistical calculation. The statistical analysis was performed using SPSS software (v. 20.0; IBM SPSS, Armonk, NY, USA).

## Results

**Fig. 1:**
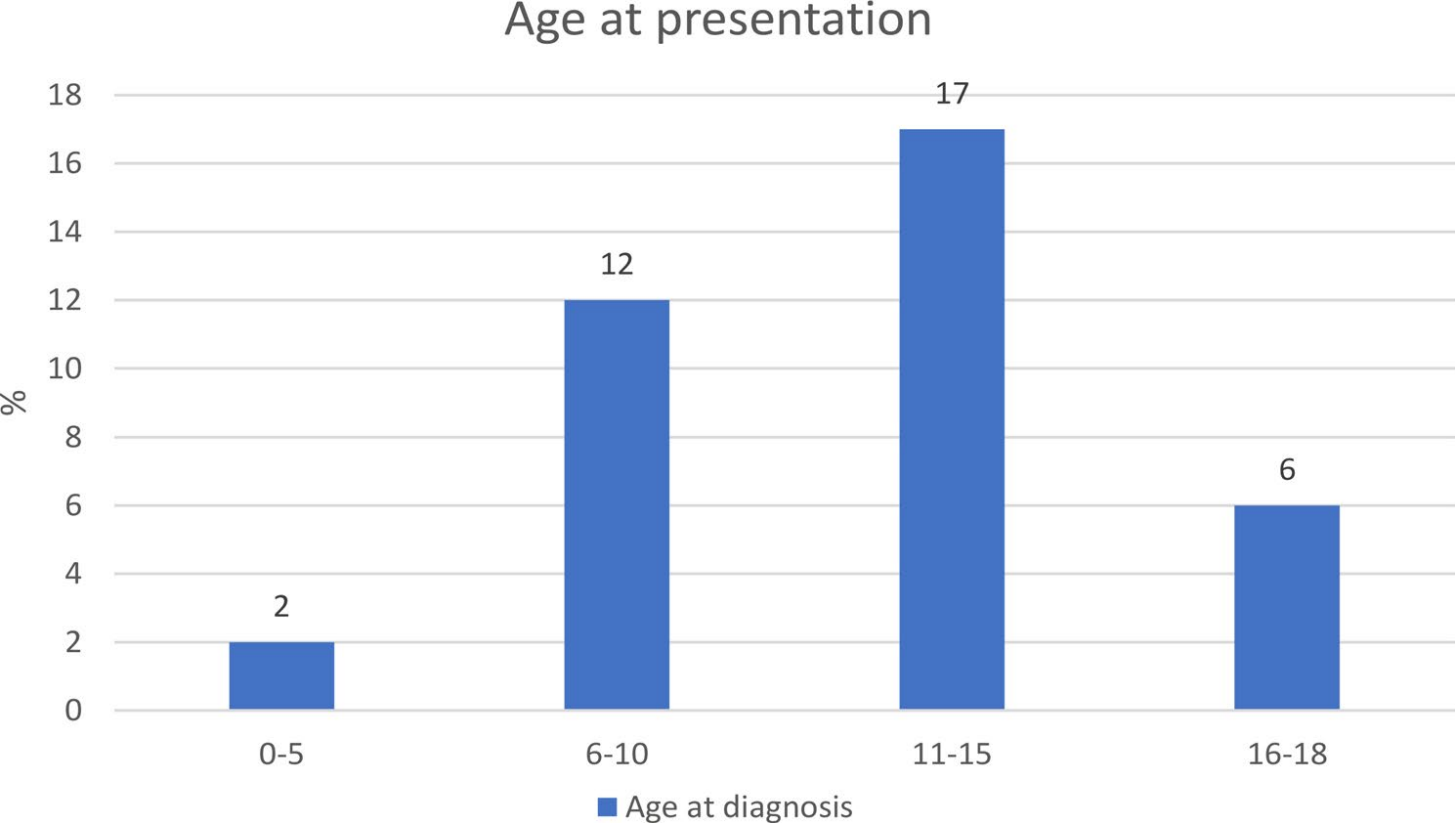
Age distribution of our cohort

**Fig. 2:**
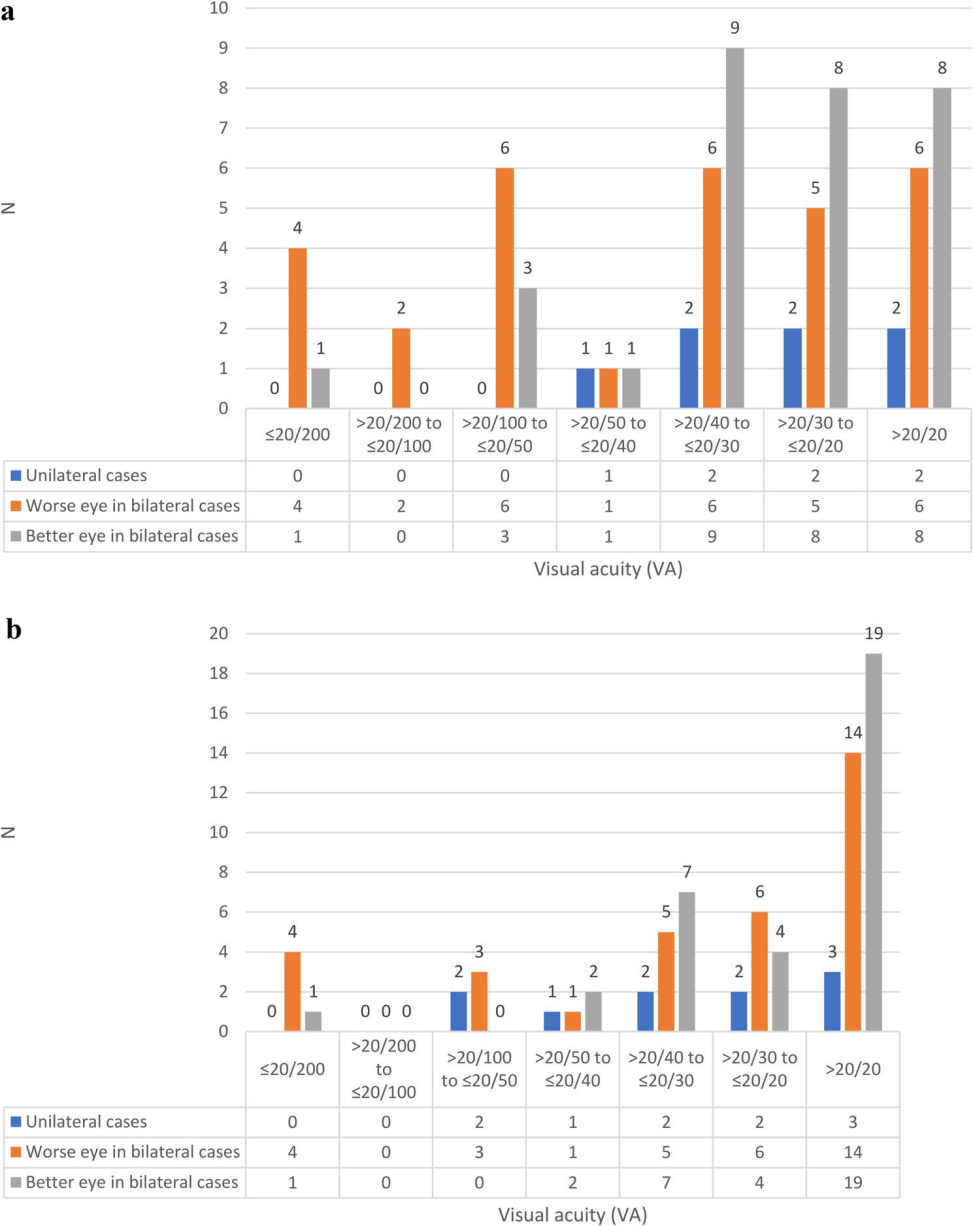
**a** Baseline visual acuity of our cohort at presentation **b** Visual acuity after treatment

A total of 37 cases of paediatric uveitis involving 67 eyes were included. The demographic information and clinical characteristics of all the recruited cases were shown in Table 3. The male to female ratio was 1:1.3. The mean age of uveitis onset was 11 ± 3.7 (4–18) (Fig. 1). 81.1% of the cases suffered from bilateral uveitis. 75.7% of the cases are chronic uveitis in terms of clinical course. Majority of the cases (40.5%) presented with anterior uveitis. The proportion of intermediate uveitis, posterior uveitis and panuveitis were 32.4%, 5.4% and 21.6% respectively. Posterior uveitis accounted for a minority of cases in our cohort. Within our study, the two instances of posterior uveitis included one case of idiopathic posterior uveitis, which presented with dense vitritis and periphlebitis, and one case of Vogt-Koyanagi-Harada disease, characterized by exudative retinal detachment. 13.5% of the uveitis cases were associated with granulomatous inflammation. Among the 5 granulomatous uveitis, only 1 case had actual diagnosis of tubulointerstitial nephritis and uveitis. 14 out of 37 cases were found to have associated systemic diseases whereas the others were idiopathic in terms of aetiology. Behçet’s disease (8.1%), tubulointerstitial nephritis and uveitis (8.1%) and HLA-B27 associated uveitis (8.1%) are seen at least as common as Juvenile Idiopathic arthritis associated uveitis (5.4%) in this cohort. A positive antinuclear antibody (ANA), defined as titre of 1:40 by indirect immunofluorescence, was observed in 29.7% of all uveitis cases. Steroid response was a common phenomenon, observed in 40.5% of cases. The most common complication was posterior synechiae (45.9%), followed by cataract (37.8%), glaucoma (27.0%), band keratopathy (18.9%) and macular oedema (13.5%). 8.1% and 13.5% of cases required glaucoma and cataract surgeries respectively. Three out of 37 patients encountered either first attack of uveitis or flare two to four weeks after receiving COVID-19 vaccine. 54.1% of patients required systemic steroid for disease control: 6 of them were severe anterior uveitis not responding to topical steroid; 6 of them were intermediate uveitis, one was posterior uveitis; and 7 of them were panuveitis. Two out of 37 cases could achieve disease control using oral steroid alone. The majority of patients who required oral steroid also needed additional steroid sparing agents for long term control, including Methotrexate (43.2%), Mycophenolate Mofetil (24.3%), Cyclosporine A (8.1%), Azathioprine (5.4%) and Tacrolimus (2.7%). 13 out of 37 cases achieved disease control with a single first line steroid sparing agent, either without oral steroid or with low dose oral steroid less than 7.5 mg per day. 11 out of 37 cases required biologics including anti-tumour necrosis factor alpha (Adalimumab 32.4%, Infliximab 2.7%) and interleukin-6 inhibitors (Tocilizumab 2.7%) in order to achieve disease control. With regard to visual acuity, the mean presenting visual acuity was 0.31 ± 0.58 logMAR and the mean visual acuity when disease was under control was 0.13 ± 0.47 logMAR. Hence, the patients in our study showed significant improvement in visual acuity when disease was controlled after treatment (*p* = 0.004). 12 out of 37 patients (5 acute uveitis, 2 recurrent uveitis, and 5 chronic uveitis respectively) were able to wean off all medications, including topical steroid, systemic steroid and systemic immunotherapy after disease control. Figure 2a and b showed the distribution of presenting visual acuity and visual acuity when uveitis under control of all eyes in terms of Snellen visual acuity. The proportion of cases with presenting visual acuity better than 20/20 was 21.6%. When uveitis was under control,45.9% of cases could reach visual acuity better than 20/20.

**Table 3:** Summary of Uveitis Characteristics

	*N*= 37	
Gender (Male: Female)		01:01.3
Male	16	43.20%
Female	21	56.80%
Mean age at presentation ±SD (range)	11	±3.7(4-18)
Clinical course		
Acute	5	13.50%
Recurrent	4	10.80%
Chronic	28	75.70%
Anatomical locations		
Anterior	15	40.50%
Intermediate	12	32.40%
Posterior	2	5.40%
Panuveitis	8	21.60%
Laterality		
Unilateral	7	18.90%
Bilateral	30	81.10%
Granulomatous inflammation	5	13.50%
Presenting VA (mean logMAR ± SD)	0.31±0.58	
VA when under disease control (mean logMAR ± SD (p value))	0.13±0.47	
	-0.004	
Aetiology		
Idiopathic	23	62.20%
Behçet’s disease	3	8.10%
Tubulointerstitial nephritis and uveitis	3	8.10%
HLA-B27 associated	3	8.10%
Juvenile idiopathic arthritis	2	5.40%
Vogt Koyanagi Harada disease	1	2.70%
Kawasaki disease	1	2.70%
Seronegative arthritis	1	2.70%
Association with autoimmune markers		
ANA	11	29.70%
ESR	2	5.40%
HLA-B27	3	8.10%
RF	1	2.70%
Steroid responder	15	40.50%
Complications		
Band keratopathy	7	18.90%
Glaucoma	10	27.00%
Posterior synechiae	17	45.90%
Cataract	14	37.80%
Macular oedema	5	13.50%
Association with COVID-19 vaccination	3	
Sinovac	1	-
BioNTech	2	-
Treatment		
Steroid		
Topical steroid	22	59.50%
Subtenon steroid	4	10.80%
Intravitreal steroid	1	2.70%
Oral steroid	20	54.10%
Steroid sparing agent		
Methotrexate	16	43.20%
Azathioprine	2	5.40%
Cyclosporine A	3	8.10%
Tacrolimus	1	2.70%
Mycophenolate mofetil	9	24.30%
Biologics		
Adalimumab	12	32.40%
Infliximab	1	2.70%
Tocilizumab	1	2.70%
Glaucoma surgery	3	8.10%
Cataract surgery	5	13.50%

## Discussion

This retrospective cohort was the first local study focusing on clinical characteristics and treatment outcomes in paediatric uveitis.

### Clinical features of paediatric uveitis

The majority of paediatric uveitis is in fact idiopathic in both Asian and Western countries. Our study also shared very similar proportion of cases in terms of gender, age of presentation, anatomical locations and laterality of paediatric uveitis with previous studies [3, 4, 6, 7, 10–16]. Chronic bilateral anterior uveitis was the most common. The aetiology of non-idiopathic uveitis varies according to the ethnic and geographic distribution [17]. Table 4 had summarized clinical features of recent studies on paediatric uveitis across different countries. Juvenile idiopathic arthritis was the most common cause in paediatric uveitis in North America and Europe [3, 4, 6, 7, 11, 13–15]. Yet, in our cohort of South East Asian, Behçet’s disease, tubulointerstitial nephritis and uveitis and HLA B27 associated uveitis were at least as common as those classical juvenile idiopathic arthritis associated uveitis. The difference in aetiology of paediatric uveitis between Caucasians and our population could be due to multifactorial causes, for example genetic, environmental and immunological factors. This requires further large controlled study to prove their associations.

**Table 4:** Comparison of recent studies on paediatric uveitis across different countries

Study	Gender predominance	Age at presentation	Common anatomical locations	Laterality	Common aetiology	Common complications
Our study	Female (56.8%)	11 (mean)	Anterior (40.5%)	Bilateral (81.1%)	1) Idiopathic (62.2%) 2) Behçet’s disease (8.1%) 3) Tubulointerstitial nephritis and uveitis (8.1%) 4) HLA B27 (8.1%)	1) Posterior synechiae (45.9%) 2) Cataract (37.8%)
Sun et al. [18] (China)	Female (50.7%)	9.0 (median)	Anterior (29.2%) Panuveitis (29.2%)	Bilateral (61.2%)	1) Idiopathic (71.3%) 2) Juvenile idiopathic arthritis (8.1%)	1) Posterior synechiae (26.1%) 2) Cataract (25.5%)
Waduthantri et al. [19] (Singapore)	Female (64.8%)	12.1 (mean)	Posterior (27.8%)	Bilateral (61.1%)	1) Idiopathic (29.6%) 2) HLA-B27 (9.3%)	1) Cataract (40.7%) 2) Glaucoma (35.2%)
Keino [10] et al. (Japan)	Female (70%)	12.9 (mean)	Anterior (56.3%)	Bilateral (81%)	1) Idiopathic (57.8%) 2) Juvenile idiopathic arthritis (17.2%)	1) Optic disc hyperaemia/oedema (40.6%) 2) Vitreous opacities (23.4%)
Shin et al. [12] (Korea)	Male (52.3%)	13 (mean)	Anterior (51.6%)	Unilateral (51.6%)	1) Idiopathic (65.2%) 2) Juvenile idiopathic arthritis (14.8%)	NA
Ferrara et a [11] (US)	Female (62.2%)	8.4 (mean)	Anterior (61.9%)	Bilateral (81.8%)	1) Idiopathic (51.4%) 2) Juvenile idiopathic arthritis (35%)	1) Cataract (43.9%) 2) Glaucoma (23.3%)
Smith et al. (US) [3]	Female (54%)	11.2 (median)	Anterior (44.6%)	Bilateral (75.7%)	1) Idiopathic (28.8%) 2) Juvenile idiopathic arthritis (20.9%)	1) Cataract (83.3%) 2) Posterior synechiae (41.7%)
Paroli et al. [15] (Italy)	Female (54.5%)	8.54 (mean)	Anterior (47.8%)	Bilateral (67.8%)	1) Juvenile idiopathic arthritis (19.9%) 2) Pars planitis (18.7%)	1) Cataract (27%) 2) Glaucoma (27%)
Kump et al. [7] (US)	Female (53.5%)	8 (mean)	Anterior (56.9%)	Bilateral (74.4%)	1) Idiopathic (58%) 2) Juvenile idiopathic arthritis (33%)	1) Cataract (41%) 2) Posterior synechiae (35%)
Rosenberg et al. [4] (US)	Female (52%)	10.4 (N/A)	Anterior (30.4%)	Bilateral (71%)	1) Idiopathic (25.8%) 2) Juvenile idiopathic arthritis (23%)	1) Posterior synechiae (54.7%) 2) Cataract (52%)
Azar et al. (Australia) [6]	Female (57.5%)	6.75 (mean)	Anterior (66%)	Unilateral (67.5%)	1) Idiopathic (60%) 2) Juvenile idiopathic arthritis (17.5%)	1) Cataract (26.4%) 2) Band keratopathy (7.5%)
Edelsten st al [16] (UK)	Female (65%)	6 (median)	Anterior (70%)	NA	1) Juvenile idiopathic arthritis (47%) 2) Idiopathic (44%)	NA
Boer et al. [13] (Netherlands)	Male (55%)	0.9 (median)	Anterior (36%)	NA	1) Idiopathic (53%) 2) Juvenile idiopathic arthritis (20%)	Cataract (35%)
Kadayifcilar et al. [14] (Turkey)	Female (51.1%)	7.6 ± 3.85 (mean)	Anterior (43.4%)	Bilateral (54.8%)	1) Idiopathic (24.2%) 2) Toxoplasmosis (21%)	Cataract (28.9%) Band keratopathy (10.9%)

### Complications of paediatric uveitis

**Fig. 3:**
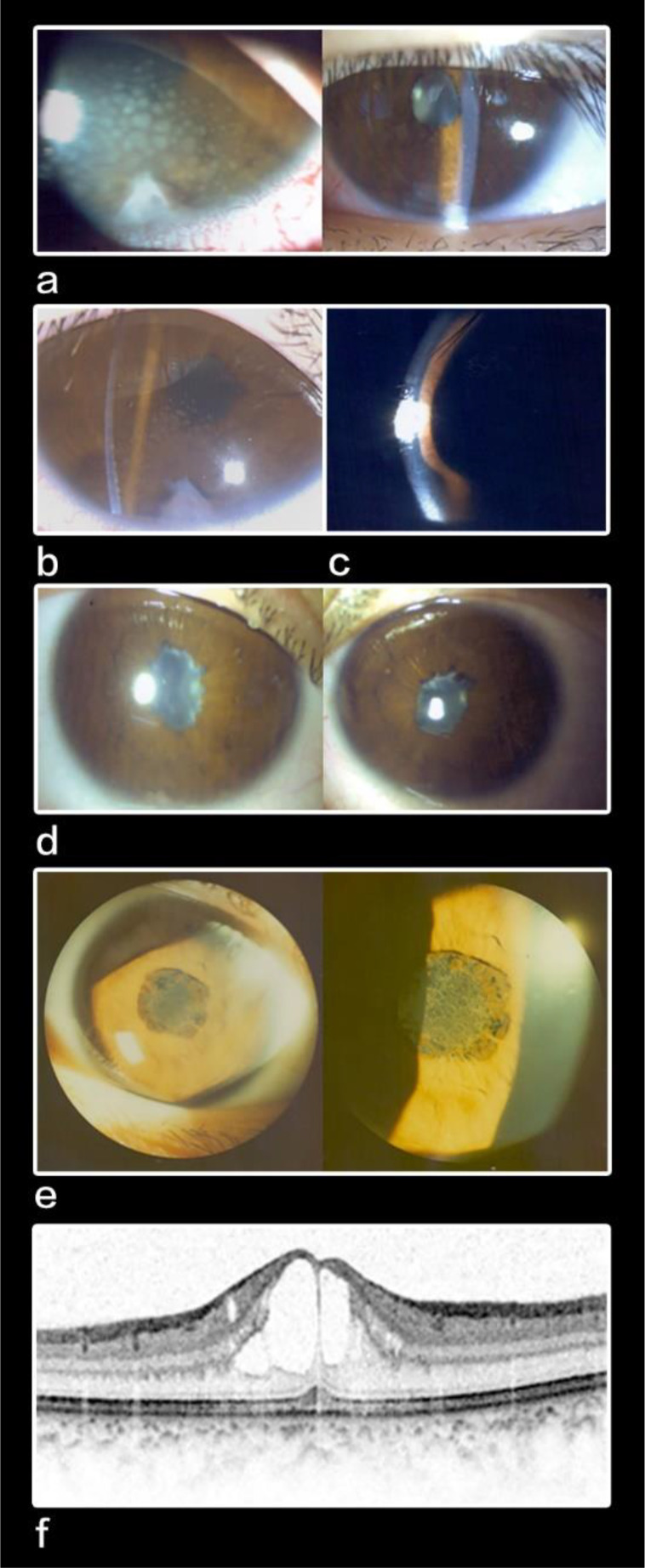
Slit lamp photos and OCT macular scans showing various clinical presentations in our cohort of paediatric uveitis **a**. A case of subacute anterior uveitis showing granulomatous keratic precipitates **b**. Slit lamp photo showing inferior broad peripheral anterior synechiae **c**. Slit lamp photo showing iris bombé **d**. Slit lamp photos of bilateral severe anterior uveitis showing 360-degree posterior synechiae **e**. Slit lamp photos showing peripapillary membrane in addition to seclusion pupillae **f**. Optical coherence tomography scan showing cystoid macular oedema

It is not uncommon for paediatric patients to develop complications after uveitis [4, 11]. Complications can arise from a delay in diagnosis and hence treatment commencement, or suboptimal control. In the literature, cataract is the most common complication, followed by glaucoma and synechiae formation in Caucasian population (Table 4). Band keratopathy is more commonly found in anterior and intermediate uveitis [4]. 26–83% of paediatric uveitis cases developed cataract [4, 6, 7, 11, 13–15, 18, 19]. Studies also reported that 35–42% of paediatric patients with uveitis patients would develop glaucoma or ocular hypertension [20], which were caused be intraocular inflammation or steroid induced. In our study, the most common complication was posterior synechiae (45.9%), followed by cataract (37.8%) and glaucoma (27.0%). Table 4 showed that both cataract and glaucoma were the commonest complications in paediatric uveitis irrespective of ethnicity. It is likely secondary to severe intraocular inflammation or use of steroid. Three of our patients received both cataract and glaucoma filtration surgeries. One of them had refractory glaucoma and thus he received multiple glaucoma surgeries including trabeculectomy, glaucoma drainage device implantation, ZEN gel stent implantation and cyclophotocoagulation. His visual acuity remained good at 20/30. Here are several examples of challenging cases of uveitis. A 12-year-old boy was diagnosed with severe idiopathic bilateral anterior uveitis with delayed presentation to us. He suffered from 360-degree posterior synechiae complicated by acute iris bombé and glaucoma (Fig. 3a and d). He had recurrent blocked laser peripheral iridotomy. Surgical lysis of posterior synechiae and surgical peripheral iridotomy were performed urgently in view of the uncontrolled secondary angle closure glaucoma. The surgical procedure was challenging as phacodonesis was noted on table and the iris was strongly adherent to the anterior lens capsule. This made the broad posterior synechiae difficult to be lysed with extreme care not to cause a breach the lens capsule. Another 9-year-old boy with Behcet’s disease suffered from extensive peripapillary membrane and seclusion pupillae bilaterally (Fig. 3e). He also required surgical lysis of posterior synechiae and surgical peripheral iridotomy. A 17-year-old girl diagnosed with bilateral idiopathic panuveitis was suffered from recurrent bilateral cystoid macular oedema (Fig. 3f). She had tried subtenon triamcinolone, intravitreal Ranibizumab and oral acetazolamide for controlling recurrent cystoid macular oedema. She was also steroid dependent despite the addition of Adalimumab and developed steroid induced glaucoma requiring trabeculectomy bilaterally.

### Treatment of paediatric uveitis

Corticosteroids alone are often insufficient to achieve remission in most cases. Early initiation of immunomodulatory therapy is recommended if inflammation persists. Steroid sparing agents and biologics play a key role to controlling ocular inflammation, maintaining disease remission, and allowing gradual tapering of steroid. Methotrexate has well established safety and efficacy profile in paediatric patients [21] and is often prescribed as first line steroid sparing agent. Mycophenolate Mofetil may be useful in paediatric uveitis although less effective in juvenile idiopathic arthritis associated uveitis [22]. The First-line Antimetabolites as Steroid-sparing Treatment (FAST) uveitis trial demonstrated that the use of Mycophenolate Mofetil, compared with methotrexate as first line corticosteroid-sparing treatment did not result in superior control of intermediate uveitis, posterior uveitis and panuveitis among young adults [23], while the efficacy may be similar in intermediate uveitis. In our study, a total of nine patients had been put on Mycophenolate Mofetil. Five of them (three intermediate uveitis, one anterior uveitis and one posterior uveitis) had achieved disease control either as monotherapy or with combination of oral steroid only. One of them was tubulointerstitial nephritis and uveitis whereas one of them was HLA B27 associated uveitis. The rest of them were idiopathic uveitis. Also, we observed that 2 of our patients could not tolerate Methotrexate because of gastrointestinal upset, mainly vomiting. Regrettably, subcutaneous form of Methotrexate was not available in our centre at the period, and hence giving kids with targeted dose of Methotrexate may cause some intolerable gastrointestinal side effects. Therefore, Mycophenolate Mofetil could be a reasonable alternative to methotrexate as first line corticosteroid-sparing treatment in paediatric uveitis in our locality.

Tumour necrosis factor alpha inhibitors and interleukin-6 inhibitors have emerged as valuable treatment options for severe or refractory paediatric cases. Studies have demonstrated their efficacy in various forms of paediatric uveitis [24–30]. Anti-tumour necrosis factor alpha was used as second line steroid sparing therapy in our study. An exception was made in the case of a 5-year-old child presenting with isolated posterior uveitis, where a rapid switch to anti-tumour necrosis factor alpha therapy was made upon recurrence during steroid tapering. The SYCAMORE trial was a randomized controlled trial of the clinical effectiveness, safety and cost effectiveness of Adalimumab in combination with Methotrexate. It demonstrated that Adalimumab, when combining with Methotrexate, resulted in a lower rate of treatment failure than Methotrexate alone [31], supporting its role in juvenile idiopathic arthritis associated uveitis.

Although steroid sparing agents are generally well-tolerated, close monitoring for potential adverse effects, such as infections and infusion reactions is essential [32]. Also, some studies have found that patients treated with Adalimumab developed neutralizing antibodies that are associated with lower serum Adalimumab trough levels and loss of clinical response [33]. Continuation of concomitant immunosuppression, such as Methotrexate, may reduce the development of anti-Adalimumab antibodies and thus maintain response to Adalimumab [34]. Still 30–40% of patients were refractory to both Methotrexate and tumour necrosis factor alpha inhibitors [35]. We acknowledge that we presumed there was no resistance to adalimumab in our cases, as each was started concurrently with either Methotrexate or Mycophenolate Mofetil at the time of adalimumab initiation. However, it is important to note that our laboratories were not equipped to test for anti-drug antibodies against adalimumab.

Our cases had shown improvement in visual acuity when the disease was under control, which is statistically significant (*p* = 0.004). 6 of our cases have used Adalimumab with Methotrexate with promising results in improving disease control. However, one of our patients suffering from juvenile idiopathic arthritis associated uveitis had persistent on and off ocular inflammation despite a total of 33-month of Adalimumab combined with Methotrexate. She also suffered from self-limiting gastrointestinal side effects from methotrexate. The disease was better controlled after switching to Tocilizumab infusion. Her presentation was compatible to resistance to both Methotrexate and Adalimumab. This suggested that the development of anti-Adalimumab antibodies was not confined to Caucasians only. Further study with collaboration of local immunologists in future might help the identification of anti-Adalimumab antibodies in our local population. Tocilizumab is a humanized anti-interleukin-6 receptor antibody. Multiple studies had shown that the use of Tocilizumab after failure of the first anti-tumour necrosis factor alpha may result in an improvement in uveitis activity of juvenile idiopathic arthritis associated uveitis and particularly responsive in reduction of cystoid macular oedema [24, 36, 37].

### Comparison with adult uveitis

Paediatric uveitis exhibit distinct clinical features and management challenges. Previous local study on Chinese patients described that paediatric-onset intermediate uveitis was found to be associated with better initial visual acuity (20/40 or better), bilateral uveitis, chronicity and the use of second line immunosuppressive agents, compared to adult-onset intermediate uveitis [38]. In terms of complication, cystoid macular oedema was less frequently found in eyes with paediatric onset intermediate uveitis. The above were all compatible with our study findings (Table 3).

One of the management challenges in paediatric uveitis was delayed presentation. Unlike adults, children might not be able to report classical symptoms like eye pain, eye redness or photophobia promptly. This might cause delay in diagnosis, treatment commencement and possibly worse visual outcome than in adult. Up to one-third of the children with uveitis are left with severely impaired vision as a result of various ocular complications [39]. Given that anterior uveitis was common in paediatric population, they might have already developed complications upon presentation to ophthalmologists, for instance 360 degree with acute iris bombé. There were difficulties in applying laser iridotomy in paediatric patients because of limited cooperation and cornea oedema when the intraocular pressure was high. Furthermore, due to high risk of steroid response in paediatric patients, treatment options like subtenon steroid should be used with great caution. Both cystoid macular oedema and glaucoma were tough to manage. The majority of our patients were able to achieve visual acuity better than 20/20 after treatment. Yet, one patient with Behcet’s disease had very poor visual acuity of hand movement only despite adequate treatment and disease control. He suffered from severe panuveitis and the visual acuity was already poor at presentation.

Paediatric uveitis tends to follow a more chronic and relapsing course compared to adult uveitis, necessitating long term management and close monitoring. It is not uncommon that conventional treatment like steroid or combination with Methotrexate are inadequate in controlling the inflammation.

## Limitations

The main drawback of our study is the limited sample size from one tertiary referral centre. Therefore, the results may not be reflecting the true epidemiology of paediatric uveitis locally. The study is retrospective in nature. Hence, there were some missing data and information bias. The presenting symptoms were not described in this study due to limitation in reporting symptoms in young children. They might only present to health care workers when the disease became severe or visual signs became more prominent that were noticed by their careers. There could be length time bias that the disease onset may be overestimated.

## Conclusion

Clinical features of paediatric uveitis locally are different from what was described in Caucasian population. In our locality, idiopathic or undifferentiated causes were the predominant reasons for paediatric uveitis, rather than Juvenile Idiopathic Arthritis associated uveitis or cases related to rheumatological conditions. According to our experience, the commonest causes of systemic associations were Behçet’s disease, tubulointerstitial nephritis and uveitis and HLA B27 associated. Our report evaluated the efficacy of immunomodulatory therapy and biologics in controlling uveitis and reducing ocular complications.

## Data Availability

The data and material are available upon reasonable request and in compliance with local data protection policy.
